# Impact of Bowel Preparation Type on Colonoscopy Quality and Adenoma Detection: A Comparative Study

**DOI:** 10.7759/cureus.82969

**Published:** 2025-04-25

**Authors:** Sankeerthan Maramraj, Elaine Yeap

**Affiliations:** 1 Gastroenterology, University Hospital Ayr, Ayr, GBR; 2 General and Colorectal Surgery, University Hospital Crosshouse, Kilmarnock, GBR

**Keywords:** adenoma detection, bowel preparation, colonoscopy, colorectal cancer screening, polyethylene glycol, sodium picosulfate

## Abstract

Background

Colorectal cancer (CRC) is a leading cause of cancer-related mortality worldwide. Colonoscopy is the gold standard for CRC screening, but its effectiveness depends on bowel preparation quality. This study compares polyethylene glycol (PEG)-based MoviPrep (Norgine Limited, Hengoed, UK) and sodium picosulfate-based Picolax (Ferring GmbH, Kiel, Germany) in terms of bowel cleansing quality, caecal and ileal intubation rates, and adenoma detection rate (ADR).

Methods

This retrospective observational study analysed 6,921 colonoscopies performed at University Hospital Crosshouse between June 2020 and June 2023. Bowel preparation quality was assessed using the modified Ottawa Bowel Preparation Scale, categorised as Excellent, Good, Fair, or Inadequate. ADR was determined by histologically confirmed adenomas. Statistical comparisons between the two groups were performed using chi-square tests.

Results

MoviPrep was used in 6,219 (89.9%) of cases, while Picolax was used in 702 (10.1%) cases. MoviPrep was associated with a lower inadequate preparation rate (343 (5.5%) vs. 63 (9.0%)), a higher caecal intubation rate (3,675 (59.1%) vs. 307 (43.7%)) and ileal intubation rate (1,119 (18.0%) vs. 81 (11.5%)), and a higher ADR (2,295 (36.9%) vs. 167 (23.8%)).

Conclusion

MoviPrep demonstrated superior bowel cleansing, higher completion rates, and greater adenoma detection, supporting its use as a preferred bowel preparation method for colonoscopy in clinical practice.

## Introduction

Colorectal cancer (CRC) remains a major global health concern, currently ranking as the third most diagnosed cancer and the second leading cause of cancer-related mortality worldwide [1]. Despite advances in treatment, early detection remains critical to improving patient outcomes. Screening programmes have proven effective in identifying precancerous lesions and reducing CRC-related mortality [2,3]. Among available screening modalities, colonoscopy is considered the gold standard due to its ability to detect and remove adenomas during the same procedure [2,3].

In Scotland, individuals aged 50-74 are invited to participate in the National Bowel Screening programme using the quantitative faecal immunochemical test (qFIT), which detects occult blood in the stool [4]. Those with a positive qFIT result are referred for urgent colonoscopy. However, the success of colonoscopy screening depends not only on the procedure itself but significantly on the quality of bowel preparation [4,5]. Inadequate bowel cleansing can obscure mucosal surfaces, leading to missed lesions, increased procedural time, and the need for repeat investigations [6].

This single-centre retrospective observational study aims to compare two widely used bowel preparation regimens, polyethylene glycol (PEG)-based MoviPrep (Norgine Limited, Hengoed, UK) and sodium picosulfate-based Picolax (Ferring GmbH, Kiel, Germany), in terms of their impact on bowel preparation quality, caecal and ileal intubation rates, and adenoma detection rates (ADRs). Through this analysis, we seek to identify the more effective preparation in routine clinical practice and contribute to optimising colonoscopy outcomes in a hospital-based setting.

## Materials and methods

Study design and setting

This retrospective observational study was conducted using data collected from procedures performed at the Endoscopy Suite of the University Hospital Crosshouse, NHS Scotland. Data were extracted from all colonoscopy procedures performed between 8 June 2020 and 11 June 2023. Ethical approval was not required, as the study involved analysis of anonymised clinical data, and no patient-identifiable information was used [7].

Study population

A total of 6,921 colonoscopies were included. Examinations without documented bowel preparation were excluded. The main variable of interest was the type of bowel preparation administered: PEG-based MoviPrep or sodium picosulfate-based Picolax.

Exclusion criteria

Undocumented bowel preparations were excluded from the analysis. Procedures that were cancelled/abandoned due to inadequate preparations or other non-preparation-related reasons were also excluded from the analysis.

Data collection

Clinical and procedural data were collected retrospectively from endoscopy reporting systems using UNISOFT's GI Reporting Tool (current version v14.70.58, UNISOFT Medical Systems, Bishops Stortford, UK). All the required information was updated into the operating system before the procedure, and the data set with the information required for the study was collected and formatted using Microsoft Excel (Microsoft Corporation, Redmond, Washington).

Dosing for MoviPrep

The two-day split-dosing regimen was considered standard. Dose 1 was administered the evening before the colonoscopy, approximately 10 to 12 hours prior, and Dose 2 was taken the next morning, on the day of the colonoscopy, approximately 12 hours after the start of Dose 1 and at least 3.5 hours prior to the colonoscopy.

Dosing for Picolax

The split-dosing regimen was considered standard, with one sachet (Dose 1) taken 10 to 18 hours before the procedure and one sachet (Dose 2) taken four to six hours before the procedure.

The collected data for the study included the type of bowel preparation used, quality of bowel preparation (subjectively assessed by the endoscopist), the completeness of examination (measured by caecal and ileal intubation rates), and adenoma/polyp detection rates based on histopathological findings. The quality of bowel preparation was graded using a modified Ottawa Bowel Preparation Scale [8], categorised as *Excellent*: small amount of clear fluid, mucosa entirely visualised (>95%); *Good*: clear fluid present, but >90% mucosa visualised; *Fair*: some soft stool/aspirate but >90% mucosa still visualised; and *Inadequate*: mucosal assessment insufficient, repeat recommended.

Colonoscopy completion was defined as successful intubation of the terminal ileum. Adenomas were defined as histologically confirmed villous/tubulovillous adenomas, high-grade dysplastic polyps, or malignancies. Data on repeat colonoscopies was not included in the analysis.

Statistical analysis

All statistical analyses were performed using IBM SPSS Statistics for Windows, Version 27 (Released 2020; IBM Corp., Armonk, New York). Descriptive statistics were used to summarise the study population and procedural characteristics. Categorical variables were reported as frequencies and percentages, including the bowel preparation quality, caecal and ileal intubation rates, and ADRs. A comparative analysis between bowel preparation groups (MoviPrep vs. Picolax) was conducted using the chi-square tests to determine the outcomes. Binary logistic regression analysis was performed for the primary outcome, adenoma detection (Yes/No), with bowel preparation type as the main independent variable. Adjusted odds ratios (aORs) with 95% confidence intervals (CIs) were reported. Subgroup analysis was conducted to assess ADR within the bowel preparation quality (*Excellent*, *Good*, *Fair*, and *Inadequate*) between the bowel preparation types. All statistical analyses had a p-value < 0.05, which was considered statistically significant.

## Results

Study cohort

A total of 6,921 colonoscopies were analysed over the three-year study period. Of these, 6,219 (89.8%) procedures were performed following preparation with PEG (MoviPrep), while 702 (10.2%) procedures utilised sodium picosulfate (Picolax).

Bowel preparation quality

The data showed that MoviPrep resulted in a lower inadequate preparation rate (5.5%) than Picolax (9.0%). The differences were statistically significant with a p-value of 0.0013. The distribution of bowel preparation quality, as assessed by the endoscopist, is presented in Table 1 [8]. Overall, MoviPrep resulted in higher rates of *Excellent*, *Good*, and *Fair* bowel preparation and a lower rate of *Inadequate* preparation, compared to Picolax.

**Table 1:** Bowel Preparation Quality by Preparation Type

Quality of Bowel Preparation	MoviPrep (n=6,219)	Picolax (n=702)
Excellent	1,743 (28.0%)	196 (27.9%)
Good	2,601 (41.8%)	293 (41.7%)
Fair	1,532 (24.6%)	150 (21.4%)
Inadequate	343 (5.5%)	63 (9.0%)

Colonoscopy completion rates

Caecal and ileal intubation rates were analysed as surrogate indicators of bowel clearance quality and procedural completeness [9,10]. As presented in Table 2, colonoscopies performed following MoviPrep preparation achieved significantly higher caecal intubation rates (59.1%) and ileal intubation rates (18.0%) compared to those following Picolax preparation (43.7% and 11.5%, respectively). The differences were statistically significant (p<0.0001), as shown in Table 4, indicating that MoviPrep facilitated better mucosal visibility and endoscopic navigation. MoviPrep significantly outperformed Picolax in both endpoints, suggesting superior bowel cleansing and improved endoscopic visibility.

**Table 2:** Colonoscopy Completion Rates by Preparation Type

Colonoscopy Completion	MoviPrep (n=6,219)	Picolax (n=702)
Caecal Intubation	3,675 (59.1%)	307 (43.7%)
Ileal Intubation	1,119 (18.0%)	81 (11.5%)

Adenoma detection rate

The overall ADR in the entire cohort was 35.6% (2,462/6,921). MoviPrep demonstrated a higher ADR (36.9%) than Picolax (23.8%), as outlined in Table 3. This difference was statistically significant (p<0.0001), supporting the conclusion that MoviPrep leads to better adenoma detection (Table 4). This is attributed to its better bowel cleansing quality, thereby aiding better mucosal visibility and increased ADRs [11].

**Table 3:** Adenoma Detection Rate by Preparation Type and Preparation Quality

Adenoma Detection	MoviPrep (n=6,219)	Picolax (n=702)
Overall ADR (n=2,462)	2,295 (36.9%)	167 (23.8%)
Excellent	620 (27%)	34 (20.3%)
Good	999 (43.5%)	83 (50%)
Fair	608 (26.5%)	41 (24.5%)
Inadequate	68 (3.0%)	9 (5.4%)

**Table 4:** Chi-Square Test Results

Outcome	Chi-Square Results	p-value	Interpretation
Bowel Preparation Quality	15.64	0.0013	Statistically significant difference
Caecal Intubation Rate	60.82	< 0.0001	Highly significant difference
Ileal Intubation Rate	21.01	< 0.0001	Highly significant difference
Adenoma Detection Rate	41.75	< 0.0001	Highly significant difference

Notably, within the *Excellent* and *Fair* preparation subgroups, ADR was consistently higher in the MoviPrep cohort, with the exception of the *Good* preparation subgroup, where Picolax had a higher rate than MoviPrep. This suggests that high-quality bowel preparation plays a crucial role in adenoma detection [12]. A small number of adenomas were also detected in reports where the bowel preparation quality was rated as *Inadequate* (MoviPrep: 68 (2.9%); Picolax: 9 (5.2%)).

Table 4 presents the results of the chi-square tests conducted to assess the statistical significance of the association between the type of bowel preparation (MoviPrep vs. Picolax) and various colonoscopy outcomes. The outcomes analysed include bowel preparation quality, caecal and ileal intubation rates, and ADRs. The results of the chi-square tests support the hypothesis that MoviPrep leads to better bowel preparation quality and improved colonoscopy outcomes compared to Picolax.

## Discussion

The rising prevalence of CRC necessitates the development and implementation of effective and efficient screening strategies that aim to detect cancers at an early stage and thereby aid with effective and timely intervention [13]. Colonoscopy continues to be the gold standard in the detection of colorectal tumours due to its high diagnostic and therapeutic capabilities [14]. However, the outcome of colonoscopy is largely dependent on the quality of bowel preparation. Inadequate bowel preparation can be associated with reduced ADRs, incomplete or abandoned examinations, prolonged procedural times due to bowel clearing/suctioning and time taken for exploring, increased procedural costs, and the necessity for repeated procedures [15].

In this retrospective observational study, a comparison was made to check for the effects of two commonly used bowel preparation types, PEG-based MoviPrep and sodium picosulfate-based Picolax, on bowel cleansing quality, ileal and caecal intubation rates, and ADRs in a single-centre, hospital-based cohort of 6,921 patients undergoing colonoscopy between June 2020 and June 2023.

The results from this study suggest that MoviPrep is superior to Picolax in multiple domains checked for the quality of colonoscopy. Firstly, regarding bowel cleansing quality, MoviPrep demonstrated a lower rate of inadequate preparations (5.5%) compared to Picolax (9%), indicating more effective bowel cleansing. This is very important, as suboptimal preparation can obscure the visual field of the colonic mucosa, potentially resulting in missed lesions and reducing the ability to complete the examination or, at times, abandoning the examination due to inadequate preparation. Though the percentage of inadequate preparation seems small, this can translate into significant numbers when considering repeated procedures and the associated costs when done over a large population. Similar studies have found that PEG-based preparation tends to provide better bowel cleansing compared to sodium-picosulfate-based preparation. For example, a study by Lee et al. demonstrated that 1 L PEG with ascorbic acid preparation achieved a high-quality cleansing rate of 87% compared to 77% with sodium picosulfate with magnesium citrate, indicating the superior efficacy of PEG-based [16].

Furthermore, MoviPrep demonstrated a higher ileal intubation rate (18%) and caecal intubation rate (59.1%) compared to Picolax (11.5% and 43.7%, respectively). These findings are significant as they emphasise the effectiveness of MoviPrep in facilitating the completion of the examination. Completion of the examination is essential, as right-sided lesions or lesions in the terminal ileum may be missed due to inadequate preparation. These findings are consistent with those of other studies, which suggest that MoviPrep provides a better colonoscopy completion rate. For example, a study by Belsey et al. demonstrated that patients prepared with prior-day dosing with PEG-based regimens had a significant advantage at colonoscopy with better proximal colon clearance than those using sodium picosulfate-based solutions [17].

The ADR serves as a very significant benchmark for the quality of colonoscopy. A higher ADR can help lower the interval for repeated procedures and increase the outcome of the procedure. The study revealed a higher overall ADR in the MoviPrep group (36.9%) compared to the Picolax group (23.8%). This difference highlights the impact of how the type of bowel preparation used can alter the outcome and help with management and clinical prognosis. This correlates with a previous study, which showed that a higher quality bowel cleansing correlates with an increased ADR, especially in the right colon using 1 L PEG [18].

Interestingly, the ADR was the highest in colonoscopies that were rated as *Good* preparations for both MoviPrep and Picolax, which correlates to the concept that optimal preparation, not necessarily excellent bowel visibility, is needed for effective polyp detection.

An interesting finding noted in the study was that adenomas were detected even in the examinations that were reported as *Inadequate*. They account for approximately 3% for MoviPrep and 5.2% for Picolax, and even though these were few, they emphasise the importance of thorough mucosal inspection, even in inadequate preparations and the potential value of biopsies taken [18]. This also highlights the necessity of repeated procedures to be done on poor or inadequate preparations to prevent missed lesions.

The findings of this study are consistent with a similar study that supports the use of PEG-based bowel preparation as being more effective in achieving a high-quality bowel prep and better outcomes [19]. While Picolax may be effective with patient tolerability due to its small volume of consumption and better taste profile, the clinical effectiveness regarding the intubation and ADR outweighs its benefits with respect to the examination [10].

The choice of bowel preparation may also be affected by the policies set up by the manufacturers, the preference of the patient, and the contraindications. For example, MoviPrep is contraindicated in patients with electrolyte imbalances, congestive heart failure, or those taking diuretics or ACE (angiotensin-converting enzyme) inhibitors [20]. Similarly, Picolax has contraindications that include significant renal impairment, severe dehydration, and inflammatory bowel disease [21]. Therefore, clinicians must balance the effectiveness, patient-specific contraindications, and tolerability when selecting an appropriate bowel preparation.

Moreover, subjective assessment tools such as the Ottawa Bowel Preparation Scale may introduce variability with the observers performing the procedure and grading the quality of the bowel preparation. Calibration and standard scoring methods across practitioners can help reduce variability and increase the reliability of future studies [22].

An additional noteworthy finding from this study is that the overall ADR was 35.6%. Although this value is slightly below the national acceptable threshold of ≥40% for high-quality colonoscopy, it still indicates a reasonably good detection performance [23]. When comparing the bowel preparation agents, MoviPrep was associated with a higher ADR at 36.9% compared to Picolax at 23.8%. The closer proximity of MoviPrep's ADR to the threshold further emphasises the diagnostic quality of colonoscopy, even though the overall cohort did not meet the benchmark. These findings align with the supporting evidence for the use of PEG-based preparations, especially in settings where maximising ADR is a priority.

Limitations

The limitations of this study include its retrospective nature, the absence of direct assessment of adenomas missed due to suboptimal prep, and insufficient data regarding patient compliance, tolerance to the bowel preparation, and the dosing or usage of adjunctive laxatives. Additionally, the quality assessment relied on the subjective judgment of the operator performing the endoscopy, which may introduce bias.

Future directions

Future studies should explore evaluating patient-centred outcomes and their experience with bowel preparation prior to the procedure, including but not limited to compliance, tolerability, dietary habits, and adjunctive laxative use. Further data on adenoma detection with segmental analysis can help compare the study with other available literature. Additionally, cost-effectiveness and strategies to improve compliance with bowel preparation protocols would be valuable in guiding clinical practice.

## Conclusions

This retrospective observational study demonstrates that PEG-based MoviPrep offers superior bowel preparation compared to picosulfate-based Picolax. Where not contraindicated, MoviPrep should be considered the preferred agent for bowel preparation to optimise colonoscopy quality and screening success. This study supports the use of PEG-based MoviPrep in most clinical scenarios, given its better and superior performance in bowel cleansing quality, intubation rates, and adenoma detection. This study also reinforces the idea that adopting an evidence-based practice can enhance the efficiency of colonoscopy services, improve/early detection of pre-malignant lesions, and contribute effectively to CRC prevention.
